# Common mental disorders and risk of female infertility: a two-sample Mendelian randomization study

**DOI:** 10.3389/fendo.2024.1433624

**Published:** 2024-09-30

**Authors:** Di Mao, Mingmei Lin, Rong Li

**Affiliations:** ^1^ Center for Reproductive Medicine, Department of Obstetrics and Gynecology, Peking University Third Hospital, Beijing, China; ^2^ National Clinical Research Center for Obstetrics and Gynecology, Peking University Third Hospital, Beijing, China; ^3^ Key Laboratory of Assisted Reproduction Ministry of Education, Peking University, Beijing, China; ^4^ Beijing Key Laboratory of Reproductive Endocrinology and Assisted Reproductive Technology, Peking University Third Hospital, Beijing, China; ^5^ Third Clinical Medical College, Peking University Health Science Center, Beijing, China

**Keywords:** mental disorders, major depressive disorder, female infertility, Mendelian randomization, genome-wide association study

## Abstract

**Introduction:**

Female infertility is a global issue that impacts on public health seriously and many mental disorders are observed in infertility groups.

**Methods:**

To investigate the casual relationship between those, genome-wide association studies summary data of anxiety disorder (n=9,897), broad depression (n=322,580), major depressive disorder (n=480,359 and n=500,199), bipolar disorder (n=51,710), insomnia (n= 462,341), and female infertility (n=126,342) were extracted from the existing datasets and was analyzed through the two-sample mendelian randomization study. The following heterogeneity and sensitivity test were applied to ensure the robustness of results.

**Results:**

Based on inverse variance weighted results, major depressive disorder was associated with female infertility (P = 0.0001, odds ratio 1.396, 95 % confidence interval 1.175–1.658). No causal relationship was identified between the other four mental disorders and infertility. was found. Additionally, reverse mendelian randomization did not indicate a causal relationship among these disorders.

**Discussion:**

The early identification and management of anxiety symptoms in women of reproductive age, in conjunction with the effective treatment of major depressive disorder, may be crucial for preserving female fertility.

## 1 Introduction

Infertility is defined by the failure to achieve pregnancy after 12 months or more of regular, unprotected sexual intercourse (1). The global incidence of infertility is on the rise due to the increasing trend of delayed marriage, childbearing, and age of childbearing, leading to a growing seriousness of the issue. According to a recent report by the World Health Organization (WHO), approximately 17.5% of adults (about 1/6 of the population) are affected by infertility, with a rising trajectory, making it a significant public health concern (2). In addition to age-related infertility, various factors such as environmental exposures, chromosomal abnormalities, lifestyle habits, and unexplained factors may also influence infertility (1). The causal relationship between mental disorders and infertility remains uncertain.

Current research indicates a higher prevalence of mental disorders among women experiencing infertility. A review of 44 studies involving 53,300 infertile female patients found rates of major depressive disorder (MDD) at 22.9%, generalized anxiety at 13.3%, stress at 78.8%, and depression at 31.6% (3). Notably, mental health complications appear to be more common among infertile women in Asia. Another study focusing on global literature from the past decade highlighted the impact of infertility on depression, anxiety, stress, and quality of life. It was observed that anxiety, depression, and decreased quality of life are common experiences for both men and women dealing with infertility (4). These findings suggest that infertility not only affects individuals physically and economically, but also significantly impacts their mental well-being, potentially leading to a range of disorders.

Many studies have identified an association between the increasing incidence of mental disorders and infertility. However, few have investigated the causal relationship and the actual impact of mental disorders on infertility as well as the reverse. Research indicates that infertile couples or women often experience higher levels of stress, leading to symptoms of depression and anxiety compared to fertile individuals (5). However, these studies were mainly observational and involved a limited sample size. Infertility can be influenced by various factors and is prone to confounding variables when examining causality. Currently, there is no definitive evidence to support the claim that mental disorders directly lead to infertility.

Based on genome-wide association studies (GWAS), mendelian randomization (MR) effectively eliminates confounding factors and distractions within the population, providing evidence to support causal associations between exposure and outcomes (6). This approach enhances the reliability of evidence for causality by using genetic variables that are independent of disease progression and reverse causality (7). The study aims to explore potential causal relationships between common mental disorders (such as anxiety disorder, broad depression, MDD, bipolar disorder, and insomnia) and female infertility through the MR method.

## 2 Materials and methods

### 2.1 Study design

**Figure 1:**
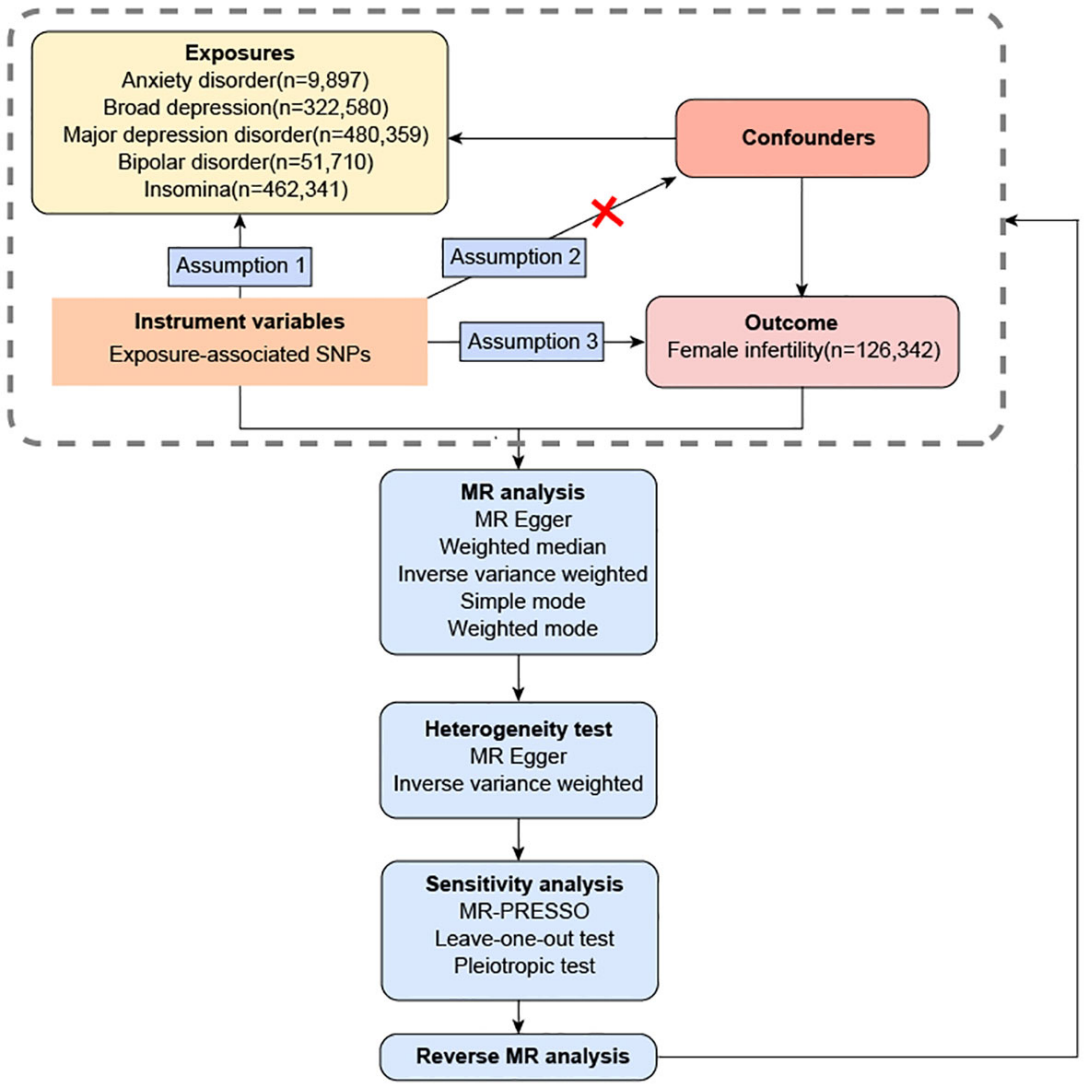
The overall flowchart of this two-sample mendelian randomization study.

Evaluation of the causal relationship between exposure (anxiety disorder, broad depression, MDD, bipolar disorder and insomnia) and outcome (female infertility) was done using the MR of two independent samples. Assumption 1 is the existence of a robust linkage between the IVs and exposure factors. Assumption 2 is that the IVs lack any association with confounding factors that might influence the relationship between exposure and outcome. Assumption 3 assumes that IVs only exert direct influence on the outcome through its association with the exposure factors. Single nucleotide polymorphisms (SNPs) were extracted from existing datasets in previous GWAS meta-analysis studies and open GWAS projects to form IVs. The population of the datasets are all from Europe, and the control group is the general population. The overview of this flowchart is shown in **Figure 1**.

### 2.2 GWAS data from mental disorders

The GWAS data on anxiety disorder (https://figshare.com/articles/dataset/panic2019/16602218), broad depression (https://datashare.ed.ac.uk/handle/10283/3036), MDD from Psychiatric Genomics Consortium [MDD (PGC), https://datashare.ed.ac.uk/handle/10283/3203], MDD from Integrative Epidemiology Unit [MDD (ieu-b-102), https://gwas.mrcieu.ac.uk/datasets/ieu-b-102/ ], bipolar disorder (https://figshare.com/articles/dataset/bip2019/14671998 ), and insomnia (https://gwas.mrcieu.ac.uk/datasets/ukb-b-3957/ ) included 9,897, 322,580, 480,359, 500,199, 51,710, and 462,341 participants, respectively (**Table 1**) (8–12). The study extracted summary data on broad depression from participants according to the self-reported responses to the question, “Have you ever seen a general practitioner/psychiatrist for nerves, anxiety, or depression?” (11) Insomnia was assessed based on the responses to the question, “Do you have trouble falling asleep at night or do you wake up in the middle of the night?” in the UK Biobank (data field: 1200) (8). All six datasets analyzed in this study were sourced from the European population. The summarized details and access to the datasets are presented in **Table 1**. It is important to note that this study solely utilized publicly available data and ethical approval was obtained from the corresponding studies.

**Table 1:** The list of Genome wide association study (GWAS) included in the mendelian randomization (MR) study.

Disease or trait	Cases	Controls	Sample size	Population	PMID or Web link
Anxiety disorder	2,137	7,760	9,897	European	https://figshare.com/articles/dataset/panic2019/16602218
Broad depression	113,769	208,811	322,580		PMID: 29662059 (https://datashare.ed.ac.uk/handle/10283/3036)
MDD (PGC)	135,458	344,901	480,359		PMID: 29700475 (https://datashare.ed.ac.uk/handle/10283/3203)
MDD (ieu-b-102)	170,756	329,443	500,199		PMID: 30718901 (https://gwas.mrcieu.ac.uk/datasets/ieu-b-102/)
Bipolar disorder	20,352	31,358	51,710		PMID: 31043756 (https://figshare.com/articles/dataset/bip2019/14671998)
Insomnia	462,341				OpenGWAS project: ukb-b-3957 (https://gwas.mrcieu.ac.uk/datasets/ukb-b-3957/)
Female infertility	14,759	111,583	126,342		gs://finngen-public-data-r10/summary_stats/finngen_R10_N14_FEMALEINFERT.gz

MDD, major depressive disorder; PGC, Psychiatric Genomics Consortium; IEU, Integrative Epidemiology Unit.

### 2.3 GWAS data from female infertility

The GWAS data on female infertility (gs://finngen-public-data-r10/summary_stats/finngen_R10_N14_FEMALEINFERT.gz) involved 126,342 participants (**Table 1**) (13). Female infertility refers to the inability to achieve pregnancy (ICD-10 N97).

### 2.4 Genetic variant selection

The selection criteria for IVs chosen from SNPs were as follows (1): A statistically significant threshold of *P*< 5 × 10^−8^ was employed for genome-wide significance to meet assumption 1. If there was no IVs left finally, the threshold of the *P*-value would extend to 5 × 10^− 6^ (2). In the clump algorithm within a 10,000 kb clump window, SNPs with linkage disequilibrium (LD) (*r^2^* > 0.001) were excluded (3). We calculated the value of R^2^ and F-statistic for each IV (14). The equations were shown below:


$$R^{2}=\frac{beta^{2}}{beta^{2}+se^{2}*N}$$

$$F=\frac{R^{2}\times \left(N-K-1\right)}{1-R^{2}}$$

N was the number of samples from the exposure GWAS. K represented the number of SNPs in IV and the value of K was 1 here because we were calculating individual SNP. R^2^ denoted the proportion of variability in iron status that can be attributed to each individual SNP. SNPs with a Cragg-Donald F-statistic<10 were excluded cause there was a weak IV bias (4, 15). Palindromic SNPs were also excluded (5). SNPs related to con-founding factors and female infertility outcomes (16) were excluded based on the LDtrait database (LDlink | An Interactive Web Tool for Exploring Linkage Disequilibrium in Population Groups (nih.gov)). Con-founding factors for female infertility included body mass index and endometriosis (17, 18).

### 2.5 MR analysis

Inverse variance weighted (IVW) analysis served as the primary method for statistical assessment of IVs and was considered the most powerful statistical analysis (19). To further validate the consistency and robustness of MR results, additional analytical techniques were employed. MR-Egger was used to detect causal relationships based on weak assumptions (Instrument Strength Independent of Direct Effect, InSIDE) (20). MR-Egger and the weighted median approach were utilized to test the MR error hypothesis arising from directional pleiotropy. Weighted median method requires genetic variables to contribute at least 50% of the total weight, effectively combining data from multiple genetic variables into a unified causal estimation. This method ensures consistency in estimation, even when up to 50% of the information comes from invalid IVs, and demonstrates a superior finite-sample type-I error rate than IVW (21). The simple mode and weighted mode method can only evaluate the causal validity according to the cluster with the largest number of SNPs, but cannot estimate the bandwidth parameter (20). If the number of SNPs is only one, the Wald ratio method is used. To address the challenge of multiple testing, a Bonferroni correction was applied, yielding a corrected *P*-value threshold of 0.01 (0.05/5). Visual representations of MR results and their stability were achieved through scatter, forest, and funnel plots.

### 2.6 Heterogeneity and sensitivity analysis

Heterogeneity was assessed through the Cochran Q-statistic by MR Egger and IVW to determine the consistency of each IV. A *P*-value<0.05 was defined as a statistically significant level in the heterogeneity test. When heterogeneity existed in the IVs, corresponding measures (such as stratified analysis) should be used to correct it and avoid the bias caused by population stratification. In addition, a multiplicative random effects model of IVW was applied when heterogeneity was present (22).

Sensitive analysis included the “leave-one-out” method, pleiotropy test and horizontal pleiotropy test. The “leave-one-out” method was used to assess the effect of individual SNP on the outcome by removing that SNP and calculating the combined effect of the remaining SNPs separately. Pleiotropy which refers to one locus exerting influence on multiple phenotypes can compromise the reliability of MR results. The MR Egger intercept was used to estimates the pleiotropic effect of a genetic variable (23). Horizontal pleiotropy was assessed by the Mendelian Randomization Pleiotropy RESidual Sum and Outlier (MR-PRESSO) global test. MR-PRESSO outlier test would specifically identify outliers for each IV and outlier IVs would be excluded if the horizontal pleiotropy was detected. Then, the MR analysis would be conducted again. This approach ensured that MR-PRESSO provided corrected causal results, free from the confounding effects of pleiotropy and outliers.

### 2.7 Reverse MR analysis

The reverse MR analysis was conducted following the above process. The female infertility was identified as exposure and the mental disorders were outcomes to further explore the causal effect of female infertility on mental disorders.

All statistical analyses were performed using the TwoSampleMR (version 0.5.11), and ggpubr packages implemented in R (version 4.3.3).

## 3 Results

### 3.1 Selection of IVs

11, 15, 33, 24, 6 and 30 SNPs associated with anxiety disorder, broad depression, MDD (PGC), MDD (ieu-b-102), bipolar disorder, and insomnia respectively were selected as IVs according to the criteria mentioned above. The detailed information of each IV is shown in **Supplementary Tables 4**-**8**.

### 3.2 Two-sample MR analysis

**Figure 2:**
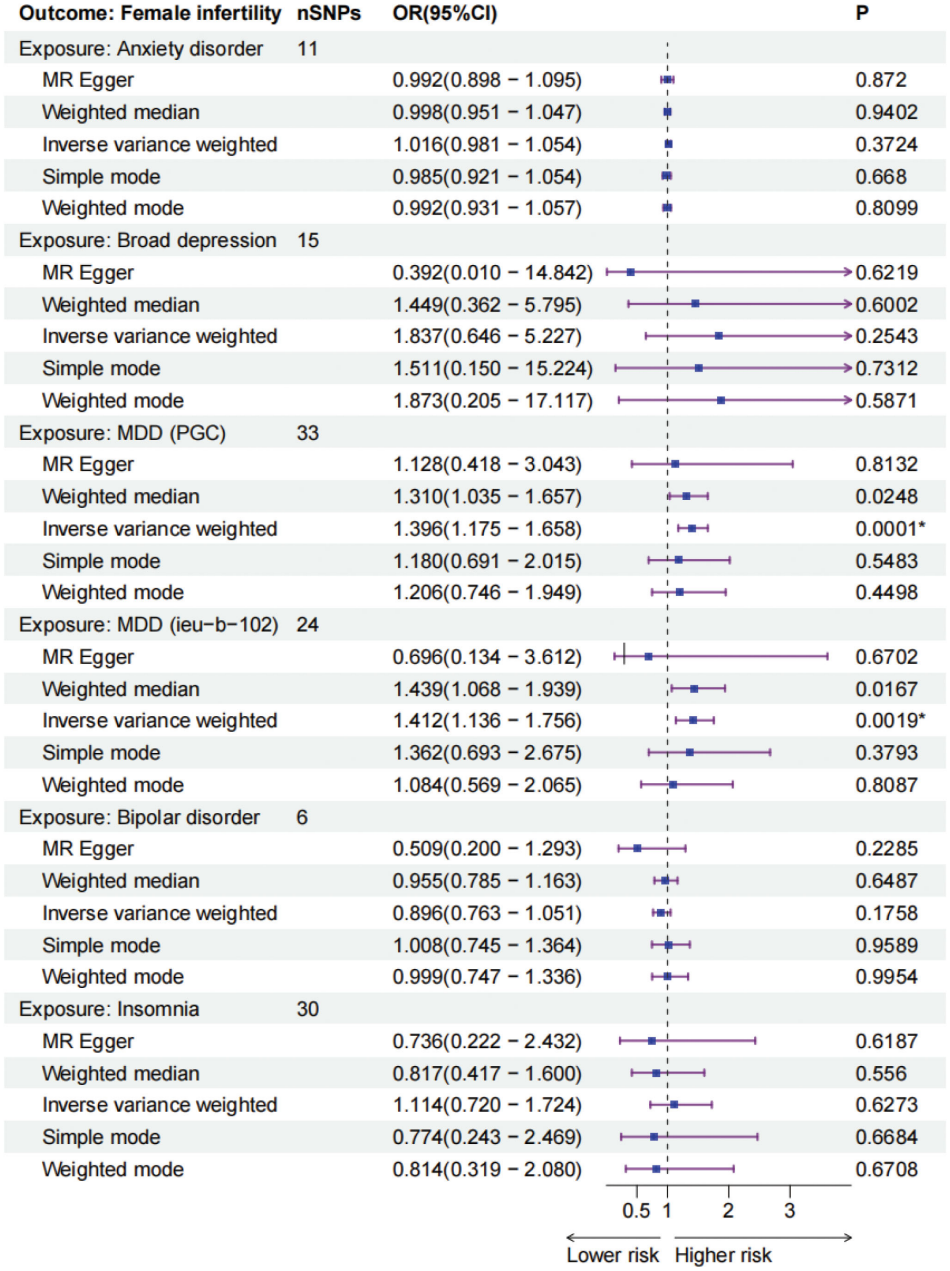
The forest plots of five mental disorders (exposures) and female infertility (outcomes). * *P*-values are ≤0.01. MDD, major depressive disorder, PGC, Psychiatric Genomics Consortium, IEU, Integrative Epidemiology Unit.

MDD (PGC) was significantly associated with an increased risk of female infertility (*P* = 0.0001, odds ratio [OR] 1.396, 95% confidence interval [CI] 1.175–1.658) based on the IVW analysis method. Additionally, MDD (ieu-b-102) produced consistent findings (*P* = 0.0019, OR 1.412, 95% CI 1.136 - 1.756). However, no causal relationship was observed between the other four mental disorders including anxiety disorder, broad depression, bipolar disorder, and insomnia, and female infertility (*P* > 0.01). The MR results for mental disorders and female infertility were shown in **Figure 2** and **Supplementary Table 2**. Scatter plots visualized the effect sizes for each MR method (**Supplementary Figure 1**), forest plots estimated the effect sizes for each SNP (**Supplementary Figure 2**), and funnel plots showed the distributions of individual SNP effects (**Supplementary Figure 3**). Results of heterogeneity test showed no statistically significant differences in the IVs both in MR Egger and IVW (**Supplementary Table 3**).

### 3.3 Sensitivity analysis

The ‘Leave-one-out’ method demonstrated that no individual SNP had a significant impact on the final results, highlighting the stability of our MR findings (**Supplementary Figure 4**). Additionally, the MR Egger pleiotropy test revealed no pleiotropic effects of instrumental variables from different exposures. Our study did not detect any horizontal pleiotropy, as indicated by the results of the MR-PRESSO global test, and outlier analysis did not identify any outlier instrumental variables (**Table 2**).

**Table 2:** The pleiotropic test of five mental disorders genetic variants in female infertility Genome-wide summary association study (GWAS) datasets.

Exposures	MR-Egger pleiotropy test			MR-PRESSO results (P)	
	Egger intercept	se	P	Global Test	Outlier Test
Anxiety disorder	0.013949	0.026715	0.614173	0.651	NA
Broad depression	0.012217	0.014044	0.400126	0.965	NA
MDD (PGC)	0.00649	0.015183	0.67201	0.2735	NA
MDD (ieu-b-102)	0.020484	0.024103	0.404575	0.2135	NA
Bipolar disorder	0.046255	0.038382	0.294584	0.4105	NA
Insomnia	0.005111	0.006995	0.471017	0.6275	NA

MDD, major depressive disorder; PGC, Psychiatric Genomics Consortium; IEU, Integrative Epidemiology Unit.

### 3.4 Reverse MR analysis

**Figure 3:**
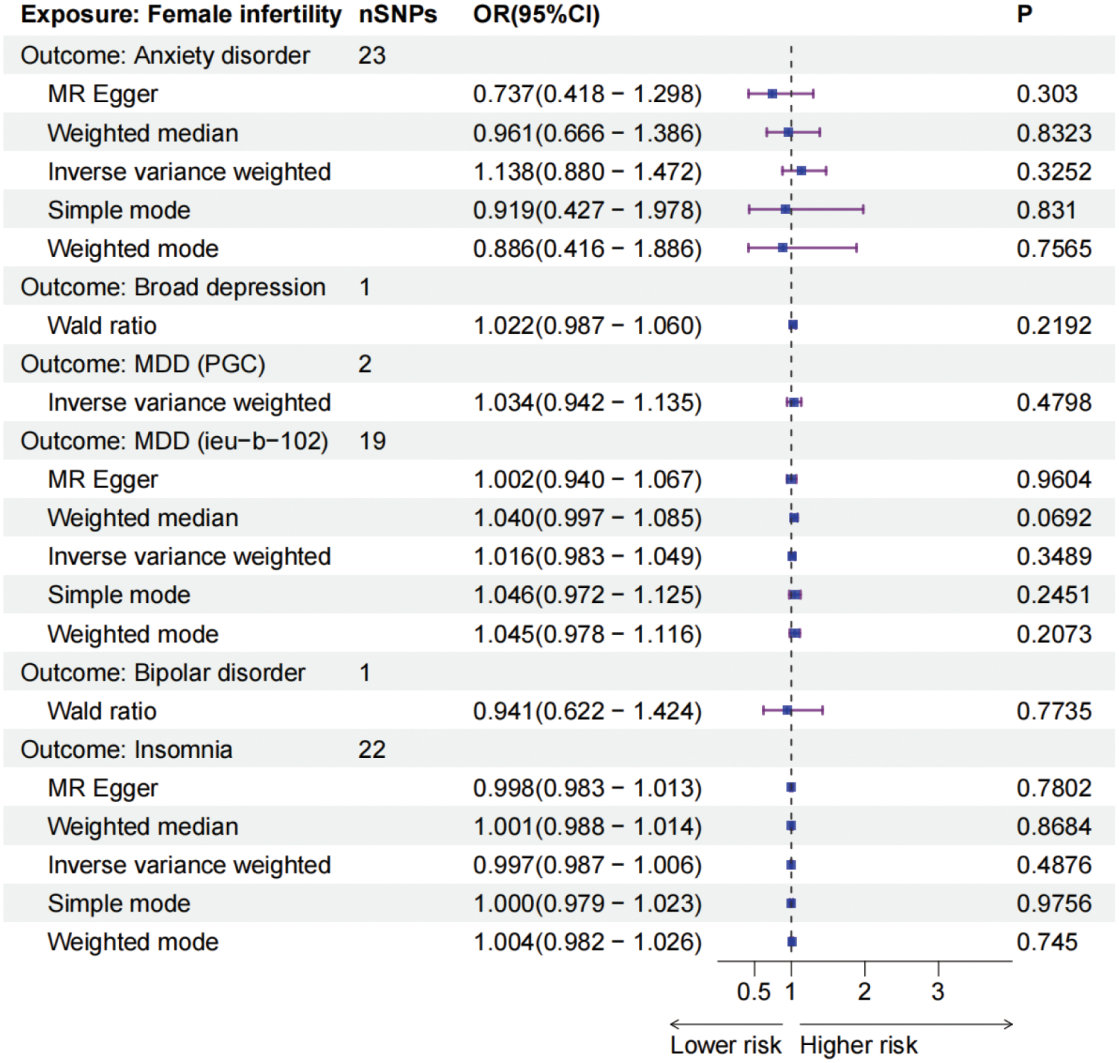
The forest plots of female infertility (outcomes) and five mental disorders (exposures). MDD, major depressive disorder, PGC, Psychiatric Genomics Consortium, IEU, Integrative Epidemiology Unit.

To further explore and ascertain the causal relationship between mental disorders and infertility, we reversed the exposure and outcome variables, treating infertility as the exposure and mental disorders as the outcome. Our analysis did not identify any causal effect of infertility on five mental disorders (**Figure 3**). Additionally, a sensitivity analysis was conducted to validate the robustness of our findings (**Table 3**; **Supplementary Table 9**-**11**).

**Table 3:** The pleiotropic test of female infertility genetic variants in five mental disorders Genome-wide summary association study (GWAS) datasets.

Outcomes	MR-Egger pleiotropy test			MR-PRESSO results (P)	
	Egger intercept	se	P	Global Test	Outlier Test
Anxiety disorder	0.040008	0.023713	0.106365	0.465	NA
MDD (ieu-b-102)	0.001399	0.002812	0.625137	0.233	NA
Insomnia	-0.00016	0.000798	0.841847	0.2145	NA

MDD, major depressive disorder; PGC, Psychiatric Genomics Consortium; IEU, Integrative Epidemiology Unit.

## 4 Discussion

Infertility is a widespread issue affecting millions of individuals of reproductive age globally, with an estimated 48 million couples facing challenges worldwide (24). The causes of infertility are multifaceted, with a growing focus on the impact of mental health factors. Previous observational studies have indicated a potential link between mental disorders and female infertility (2, 25). Research has shown that women experiencing conception difficulties are twice as likely to suffer from emotional disorders compared to fertile women (26). However, the reliability of these findings is limited by small sample sizes and potential biases in the observational studies. Therefore, caution is advised when interpreting these results, and further research is needed to confirm and build upon these initial observations. Our study found a significant association between MDD and the risk of infertility in the main analysis (OR = 1.396, *P* = 0.0001 and OR = 1.412, *P* = 0.0019) from various data sources to further validate the robustness of our findings. However, no relationship between anxiety disorder, broad depression, bipolar disorder, insomnia, and female infertility was observed in our analysis. The reverse MR analysis did not indicated the causal effect of infertility on these mental disorders. These findings provide valuable insights into the intricate relationship between specific psychiatric conditions and female infertility, potentially leading to better education for women and well-informed recommendations.

The MR model was developed utilizing aggregated data from the GWAS, leveraging its strengths in terms of the extensive sample size thus ensuring a high statistical power for our estimations. To mitigate the risk of population stratification, we imposed strict inclusion criteria, limiting our analysis to individuals of European descent. Furthermore, we focused exclusively on data from the exposed and normal populations, meticulously excluding the potential biases arising from other diseases. To enhance the scope of our analysis, we broadened the P-value threshold, enabling the inclusion of a wider array of IVs related to anxiety disorder, broad depression and bipolar disorder. Various data sources of MDD were recruited for validation. Sensitivity tests and heterogeneity assessments further corroborated the robustness of our MR findings. Notably, traditional observational studies often encounter confounding factors, such as embryological defects, immunological issues, and environmental factors, which can complicate the relationship between mental disorders and female infertility. Our study, however, offered a novel approach, exploring the risk of infertility based on the levels of mental exposure factors determined by genetic variables. Furthermore, the study excluded reverse causality, crucial in exploring infertility among women predisposed to these mental disorders. The reverse MR was also utilized to further examine the causal effect of infertility on mental disorders. MDD often necessitates medication, while broad depression is more common among infertile women. Investigating the impact of varying depression levels on infertility risk is vital. Our findings suggest MDD can lead to female infertility, while broad depression showed no significant association. Our dataset lacked MDD treatment data, reducing the likelihood of infertility due to antidepressants.

MDD is a severe mental illness characterized by symptoms such as depression, loss of interest, decreased energy, and potential suicidal thoughts or behaviors. This emotional state not only impacts daily life and work but can also have negative effects on reproductive health. A meta-analysis found that the prevalence of depression symptoms in infertile women ranged from 21% to 52.2% (27). Research indicates that individuals with severe depressive states have a higher infertility rate compared to the general population (28). Depression can disrupt the hypothalamic-pituitary-ovarian axis, affecting follicular development and ovulation (29). Additionally, individuals with severe depression may lack a positive attitude and motivation towards life, leading to a decline in sexual quality and impacting conception success rates. A cross-sectional study has revealed an association between MDD and risky sexual behaviors, including having more than ten lifetime partners (30). A MR study identified MDD as a risk factor that lowers the age of first sexual intercourse and increases the number of sexual partners over a lifetime (31). Research has found that reported condom use is lower among individuals with depression (32). These results suggest that MDD may not contribute to female infertility by reducing the frequency of unprotected sexual intercourse. Furthermore, MDD often necessitates long-term medication treatment, which distinguishes it from general depression commonly observed in infertile women. Thus, the potential mechanisms through which MDD leads to female infertility and the role of antidepressants in this causal relationship warrant further investigation. It is crucial to explore the varying impacts of different levels of depression on female infertility. Our study revealed a direct causal relationship between MDD and infertility, while no causal link was found between general depression and infertility.

Anxiety disorder is a prevalent mental health condition characterized by emotional symptoms such as excessive worry, nervousness, and fear. Numerous studies have indicated a correlation between anxiety and female infertility (33, 34). Individuals with anxiety disorders may experience endocrine disruptions as a result of prolonged and intense mental stress, ultimately impacting the normal functioning of the reproductive system. Research suggests that individuals dealing with infertility often face reduced quality of life and heightened levels of anxiety (35, 36). Particularly, studies have shown higher levels of anxiety among women struggling with infertility (37–39), with a substantial 67% of infertile patients reportedly experiencing anxiety (40). However, our study did not establish a causal relationship between anxiety disorders and female infertility.

Bipolar disorder is a mental illness characterized by both manic and hypomanic, as well as depressive episodes. These emotional fluctuations can have a significant impact on mental health and may also affect reproductive health. Recent research has shown that 24.7% of couples where one partner has bipolar disorder were childless after four years of marriage, which is approximately 13 times higher prevalence of infertility compared to national data (41). However, there is no consensus on the association between bipolar disorder and female infertility. Studies have shown conflicting results between hospitalized individuals and the general population (42). Some studies have found that patients with bipolar disorder have lower fertility rates compared to those with MDD (43, 44). Our study found no significant association between bidirectional emotional states in bipolar disorder and female infertility.

Insomnia, a common sleep disorder, can have negative effects on female reproductive health and pregnancy outcomes by impacting sleep quality, duration, and hormonal circadian rhythms (45, 46). Research has shown a significant connection between sleep disorders and female infertility, even after considering various factors like age, race, education, BMI, and lifestyle habits. Individuals with sleep disorders had a 2.14-fold higher risk of infertility compared to those without (47). Disrupted circadian rhythms in females may affect oocyte quality and quantity, while dysregulation of steroid hormones during embryo implantation could also contribute to infertility (48, 49). Insomnia may alter the expression of circadian genes, leading to cell activity timing issues and potentially increasing the risk of infertility. However, a meta-analysis of observational studies did not find a direct link between sleep disorders and infertility risk, which aligns with our study’s conclusion that insomnia is not a causal factor for infertility.

The debate regarding the potential link between infertility and the risk of mental disorders remains ongoing. Numerous studies have reported elevated incidences of anxiety, depression, sleep disorders, and other mental health issues among individuals experiencing infertility (3, 47, 50). However, our research findings suggest that the infertility phenotype does not increase the risk of developing mental disorders. This may imply that mental disorders in infertile patients could originate earlier, possibly even before the diagnosis or onset of infertility, or that they are primarily influenced by various external factors, such as social stress. Previous MR studies have produced conflicting results concerning whether female infertility heightens the risk of bipolar disorder (51, 52). In contrast to earlier MR analyses, our study employs updated GWAS data for bipolar disorder phenotypes, reinforcing the conclusion that infertility does not elevate the risk of bipolar disorder. Nevertheless, these findings necessitate further validation through multi-center randomized controlled trials.

This study aimed to investigate the causal relationship between mental disorders and female infertility. The findings did not support a causal link between anxiety disorder, broad depression, bipolar disorder, and insomnia with infertility and the causal effect of female infertility on mental disorders. Consequently, it is crucial to address the psychological well-being and mental stress of women of reproductive age. Early identification of those at risk for anxiety is vital to prevent the progression to MDD, which can negatively impact female fertility. Additionally, for women of reproductive age diagnosed with MDD, proactive management and treatment are essential to safeguard and preserve their fertility. However, a causal relationship was observed between MDD and infertility. Limitations of the study include (1): focusing only on the European population, which may impact the generalizability of the results due to genetic variations among ethnic groups; (2) lack of analysis on the duration of mental disorders due to unavailable information; and (3) challenges in fully detecting genetic pleiotropy. Future research should consider expanding the sample size, enhancing research methodologies, delving into the underlying mechanisms, and proposing more effective intervention strategies. Collaboration across disciplines such as psychology, reproductive medicine, and neuroscience is crucial for making significant advancements in understanding the relationship between mental illness and infertility.

## Data Availability

The original contributions presented in the study are included in the article/**Supplementary Material**, further inquiries can be directed to the corresponding author/s.

## Supplementary material

Supplementary Figure 1 The scatter plots of mendelian randomization when exposure are mental disorders and outcome is female infertility. (A)-(F) refers the scatter plot of each mental disorders including anxiety disorder, broad depression, MDD (PCG), MDD (ieu-b-102), bipolar disorder and insomnia. MDD, major depressive disorder; PGC, Psychiatric Genomics Consortium; IEU, Integrative Epidemiology Unit.

Supplementary Figure 2 The forest plots for each SNP of mendelian randomization when exposure are mental disorders and outcome is female infertility. (A)-(F) refers the scatter plot of each mental disorders including anxiety disorder, broad depression, MDD (PCG), MDD (ieu-b-102), bipolar disorder and insomnia. MDD, major depressive disorder; PGC, Psychiatric Genomics Consortium; IEU, Integrative Epidemiology Unit.

Supplementary Figure 3 The funnel plots for of mendelian randomization when exposure are mental disorders and outcome is female infertility. (A)-(F) refers the scatter plot of each mental disorders including anxiety disorder, broad depression, MDD (PCG), MDD (ieu-b-102), bipolar disorder and insomnia. MDD, major depressive disorder; PGC, Psychiatric Genomics Consortium; IEU, Integrative Epidemiology Unit.

Supplementary Figure 4 The results of ‘leave-one-out’ of mendelian randomization when exposure are mental disorders and outcome is female infertility. (A)-(F) refers the scatter plot of each mental disorders including anxiety disorder, broad depression, MDD (PCG), MDD (ieu-b-102), bipolar disorder and insomnia. MDD, major depressive disorder; PGC, Psychiatric Genomics Consortium; IEU, Integrative Epidemiology Unit.

Supplementary Figure 5 The scatter plots of mendelian randomization when exposure is female infertility and outcome are mental disorders. (A)-(D) refers the scatter plot of each mental disorders including anxiety disorder, MDD (PCG), MDD (ieu-b-102), and insomnia. MDD, major depressive disorder; PGC, Psychiatric Genomics Consortium; IEU, Integrative Epidemiology Unit.

Supplementary Figure 6 The forest plots for each SNP of mendelian randomization when exposure is female infertility and outcome are mental disorders. (A)-(D) refers the scatter plot of each mental disorders including anxiety disorder, MDD (PCG), MDD (ieu-b-102), and insomnia. MDD, major depressive disorder; PGC, Psychiatric Genomics Consortium; IEU, Integrative Epidemiology Unit.

Supplementary Figure 7 The funnel plots for of mendelian randomization when exposure is female infertility and outcome are mental disorders. (A)-(D) refers the scatter plot of each mental disorders including anxiety disorder, MDD (PCG), MDD (ieu-b-102), and insomnia. MDD, major depressive disorder; PGC, Psychiatric Genomics Consortium; IEU, Integrative Epidemiology Unit.

Supplementary Figure 8 The results of ‘leave-one-out’ of mendelian randomization when exposure is female infertility and outcome are mental disorders. (A)-(C) refers the scatter plot of each mental disorders including anxiety disorder, MDD (ieu-b-102), and insomnia. MDD, major depressive disorder; IEU, Integrative Epidemiology Unit.
