# Supplementary figures and images for: Common mental disorders and risk of female infertility: a two-sample Mendelian randomization study
Source: Front Endocrinol (Lausanne). 2024 Sep 30;15:1433624. doi: 10.3389/fendo.2024.1433624 (PMC11471633; doi:10.3389/fendo.2024.1433624)

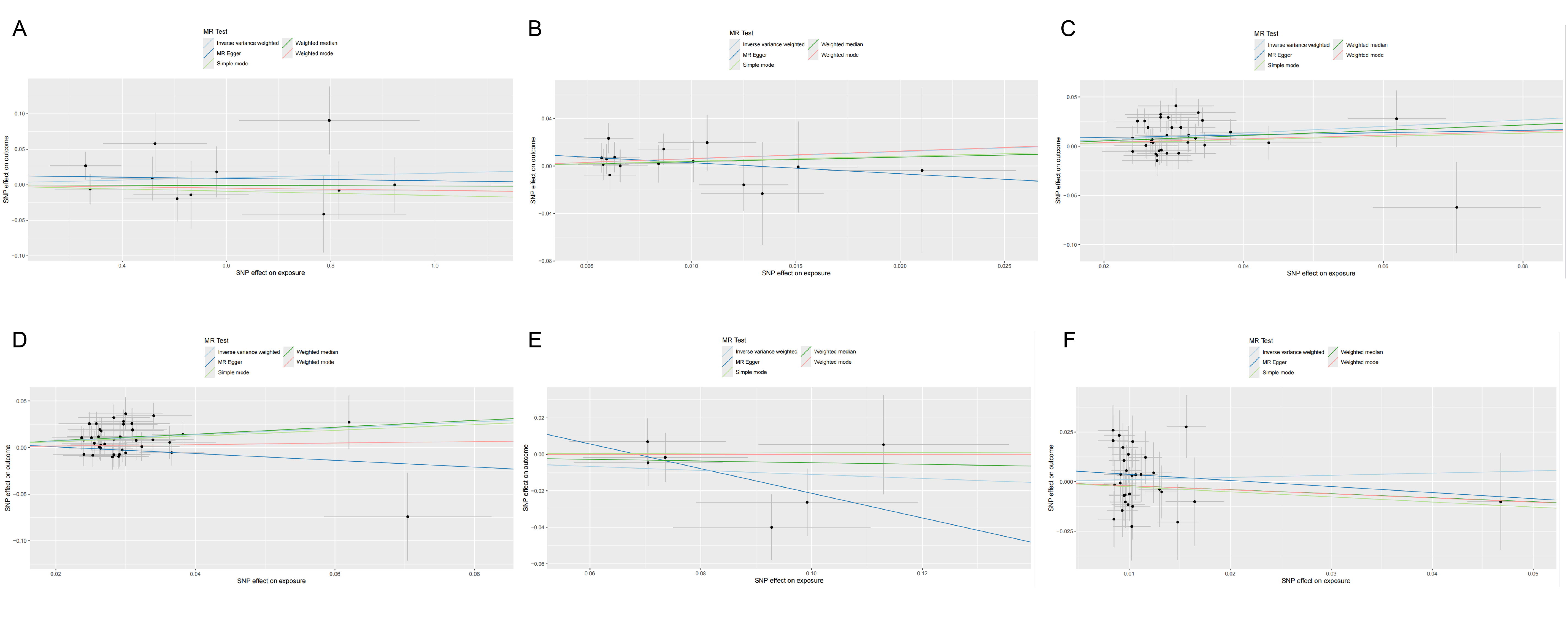

Supplement: Supplementary Figure 1 — The scatter plots of mendelian randomization when exposure are mental disorders and outcome is female infertility. (A)-(F) refers the scatter plot of each mental disorders including anxiety disorder, broad depression, MDD (PCG), MDD (ieu-b-102), bipolar disorder and insomnia. MDD, major depressive disorder; PGC, Psychiatric Genomics Consortium; IEU, Integrative Epidemiology Unit. [file Image1.png]

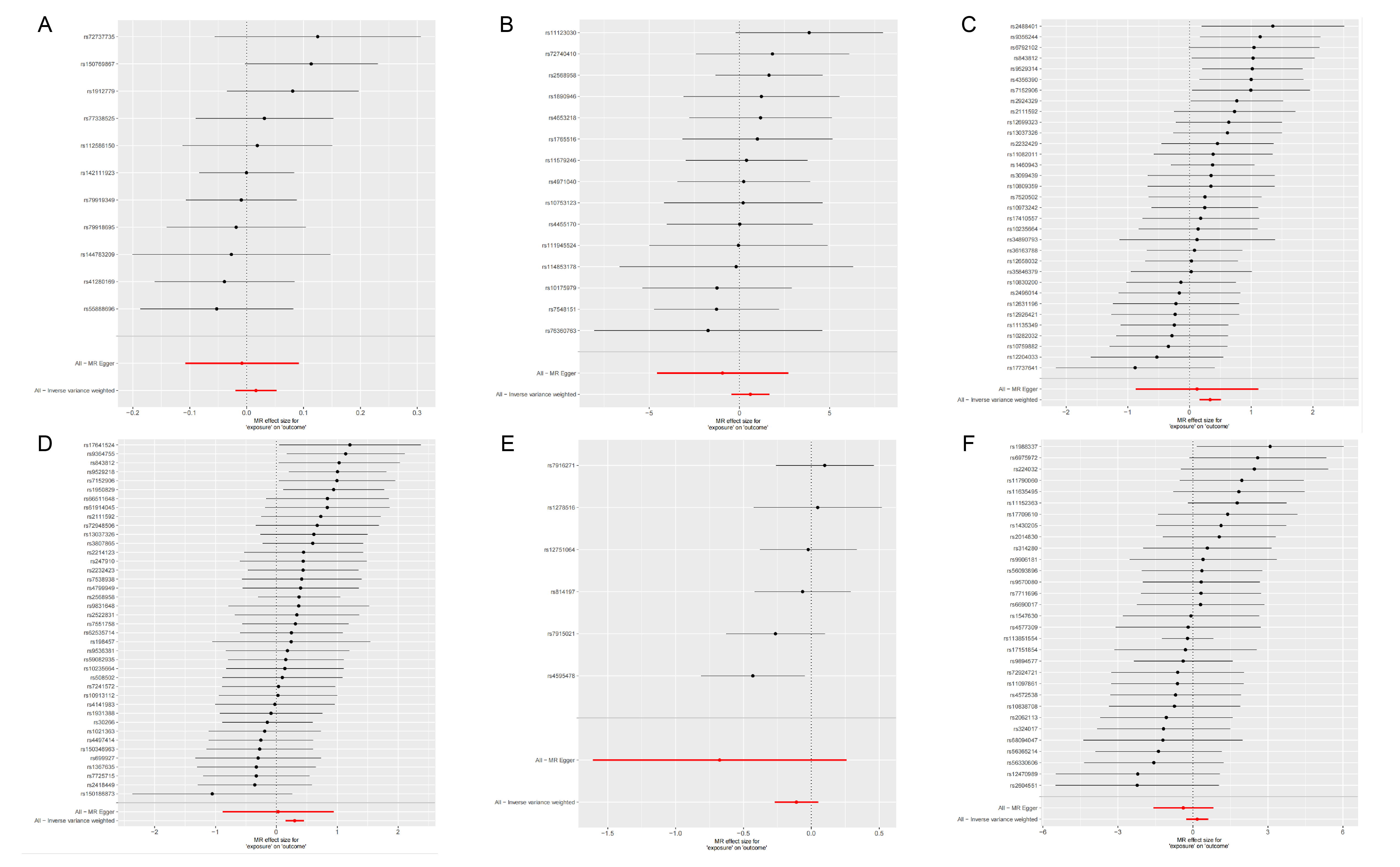

Supplement: Supplementary Figure 2 — The forest plots for each SNP of mendelian randomization when exposure are mental disorders and outcome is female infertility. (A)-(F) refers the scatter plot of each mental disorders including anxiety disorder, broad depression, MDD (PCG), MDD (ieu-b-102), bipolar disorder and insomnia. MDD, major depressive disorder; PGC, Psychiatric Genomics Consortium; IEU, Integrative Epidemiology Unit. [file Image2.png]

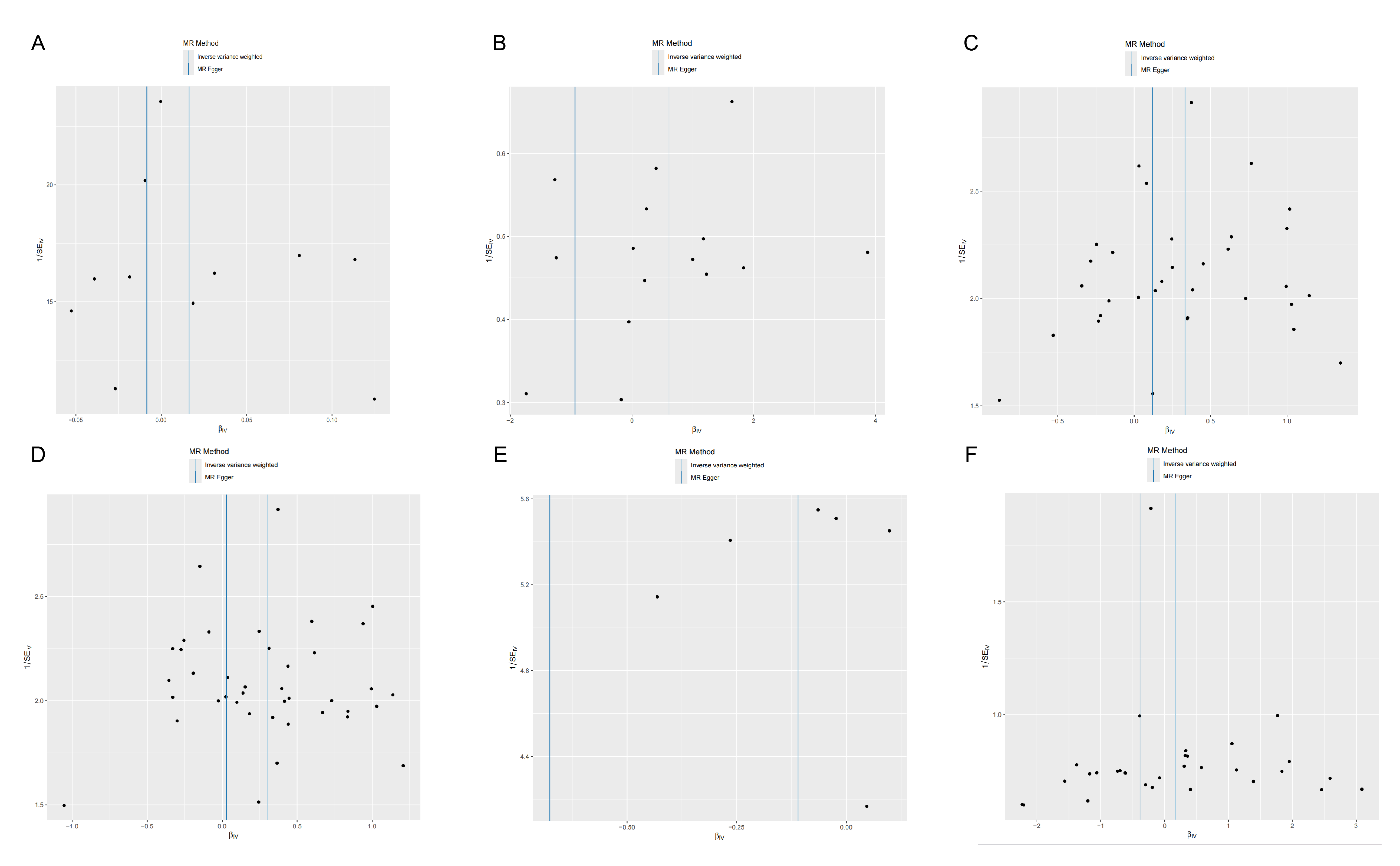

Supplement: Supplementary Figure 3 — The funnel plots for of mendelian randomization when exposure are mental disorders and outcome is female infertility. (A)-(F) refers the scatter plot of each mental disorders including anxiety disorder, broad depression, MDD (PCG), MDD (ieu-b-102), bipolar disorder and insomnia. MDD, major depressive disorder; PGC, Psychiatric Genomics Consortium; IEU, Integrative Epidemiology Unit. [file Image3.png]

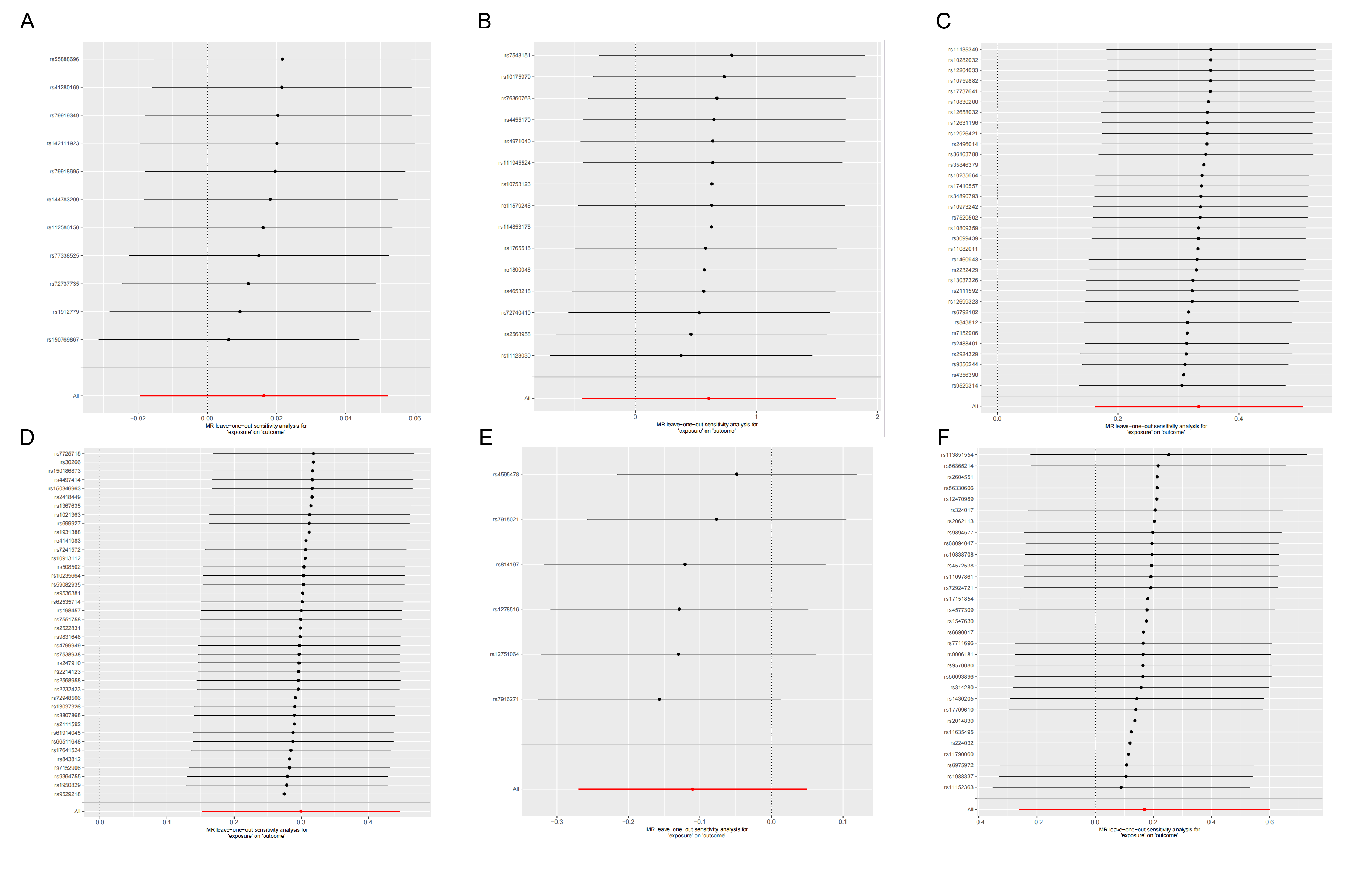

Supplement: Supplementary Figure 4 — The results of ‘leave-one-out’ of mendelian randomization when exposure are mental disorders and outcome is female infertility. (A)-(F) refers the scatter plot of each mental disorders including anxiety disorder, broad depression, MDD (PCG), MDD (ieu-b-102), bipolar disorder and insomnia. MDD, major depressive disorder; PGC, Psychiatric Genomics Consortium; IEU, Integrative Epidemiology Unit. [file Image4.png]

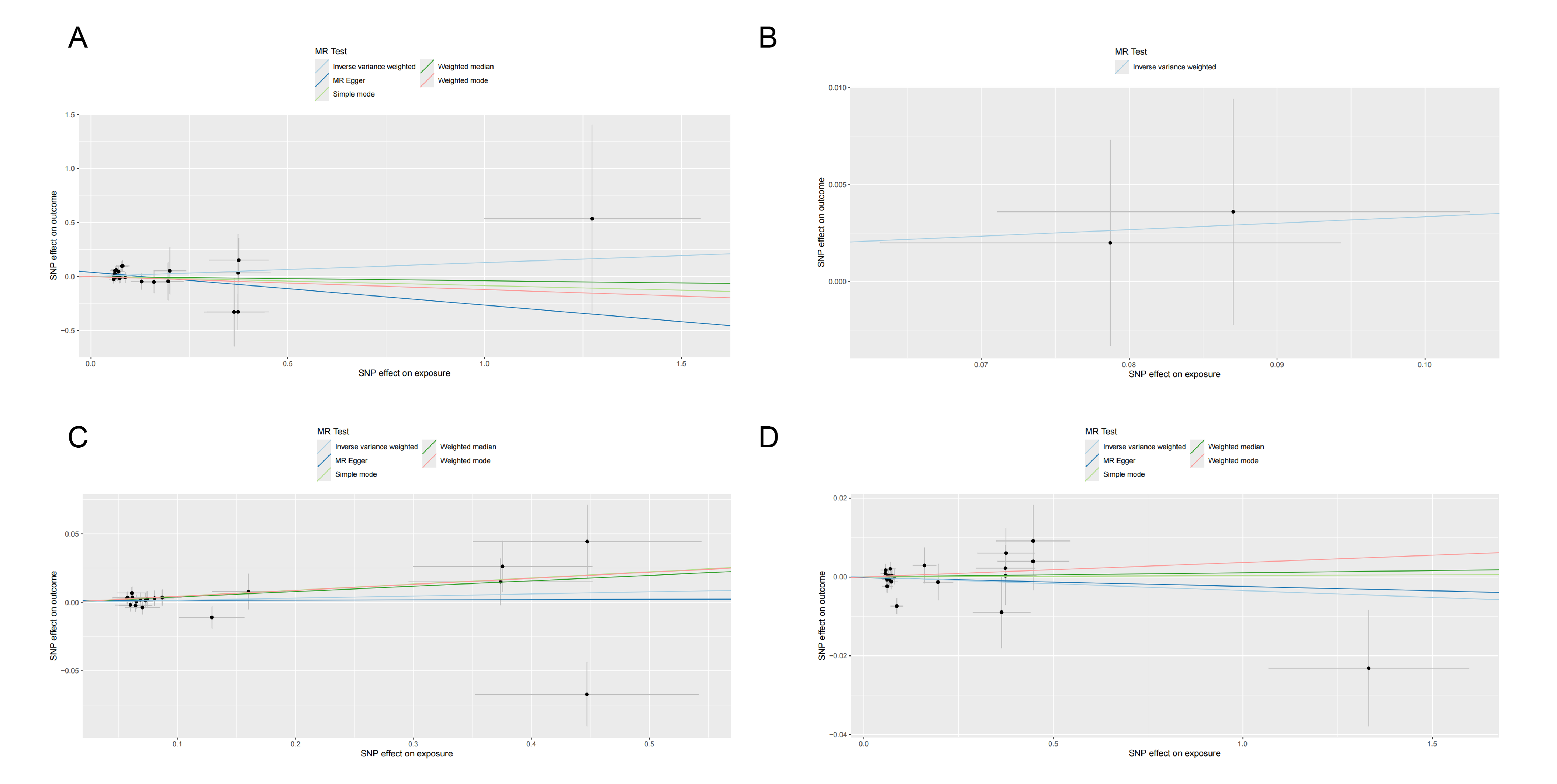

Supplement: Supplementary Figure 5 — The scatter plots of mendelian randomization when exposure is female infertility and outcome are mental disorders. (A)-(D) refers the scatter plot of each mental disorders including anxiety disorder, MDD (PCG), MDD (ieu-b-102), and insomnia. MDD, major depressive disorder; PGC, Psychiatric Genomics Consortium; IEU, Integrative Epidemiology Unit. [file Image5.png]

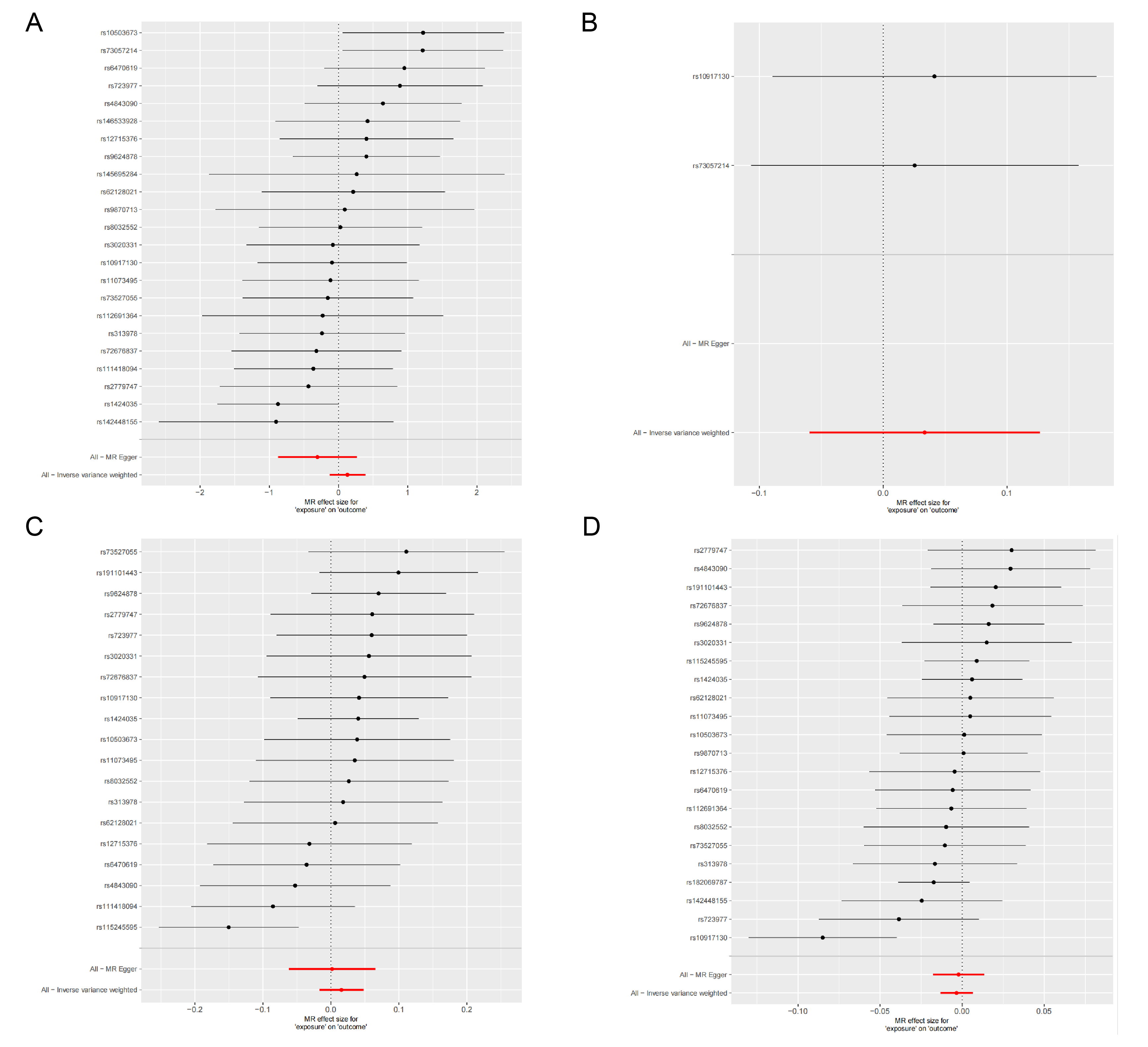

Supplement: Supplementary Figure 6 — The forest plots for each SNP of mendelian randomization when exposure is female infertility and outcome are mental disorders. (A)-(D) refers the scatter plot of each mental disorders including anxiety disorder, MDD (PCG), MDD (ieu-b-102), and insomnia. MDD, major depressive disorder; PGC, Psychiatric Genomics Consortium; IEU, Integrative Epidemiology Unit. [file Image6.png]

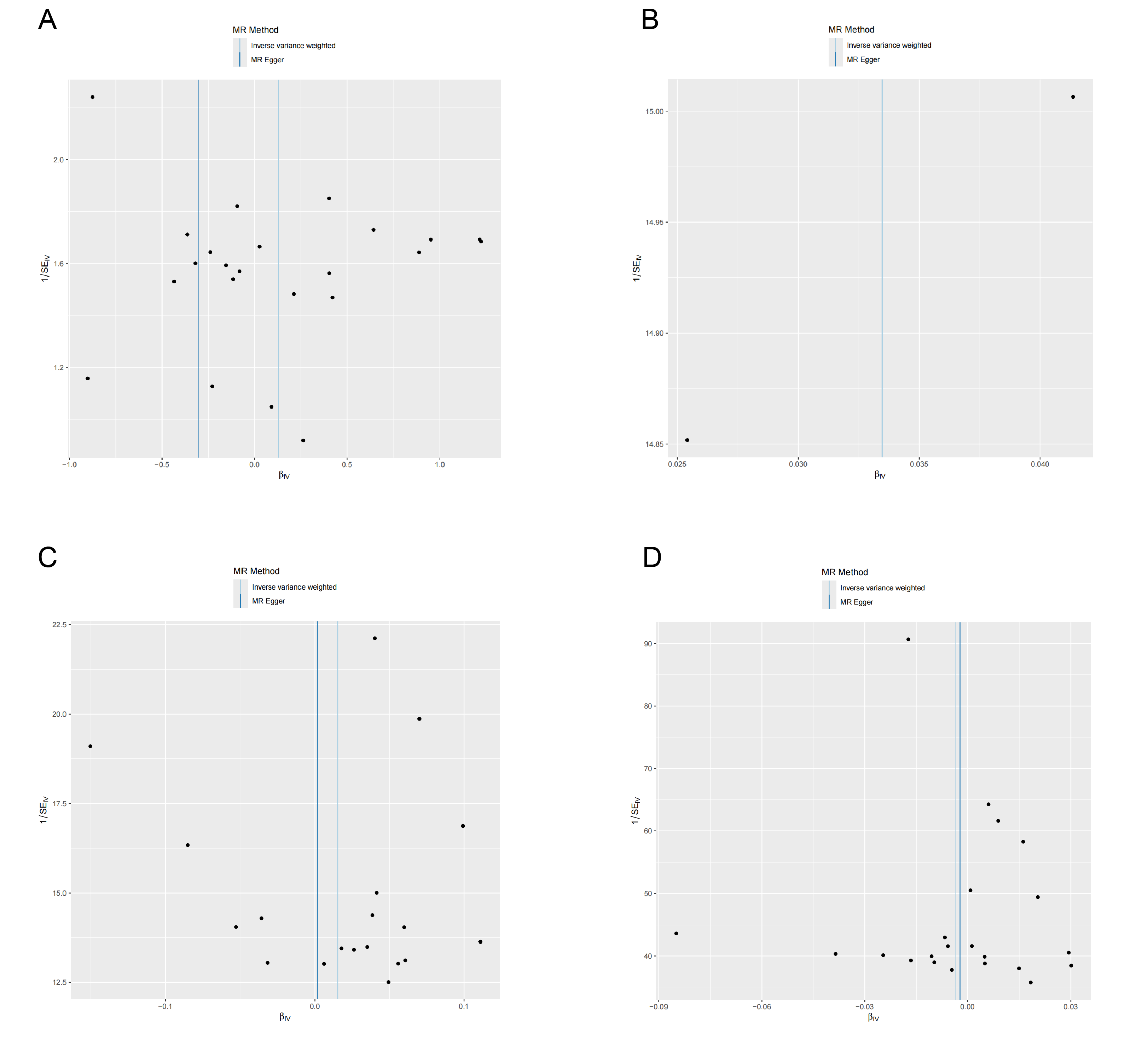

Supplement: Supplementary Figure 7 — The funnel plots for of mendelian randomization when exposure is female infertility and outcome are mental disorders. (A)-(D) refers the scatter plot of each mental disorders including anxiety disorder, MDD (PCG), MDD (ieu-b-102), and insomnia. MDD, major depressive disorder; PGC, Psychiatric Genomics Consortium; IEU, Integrative Epidemiology Unit. [file Image7.png]

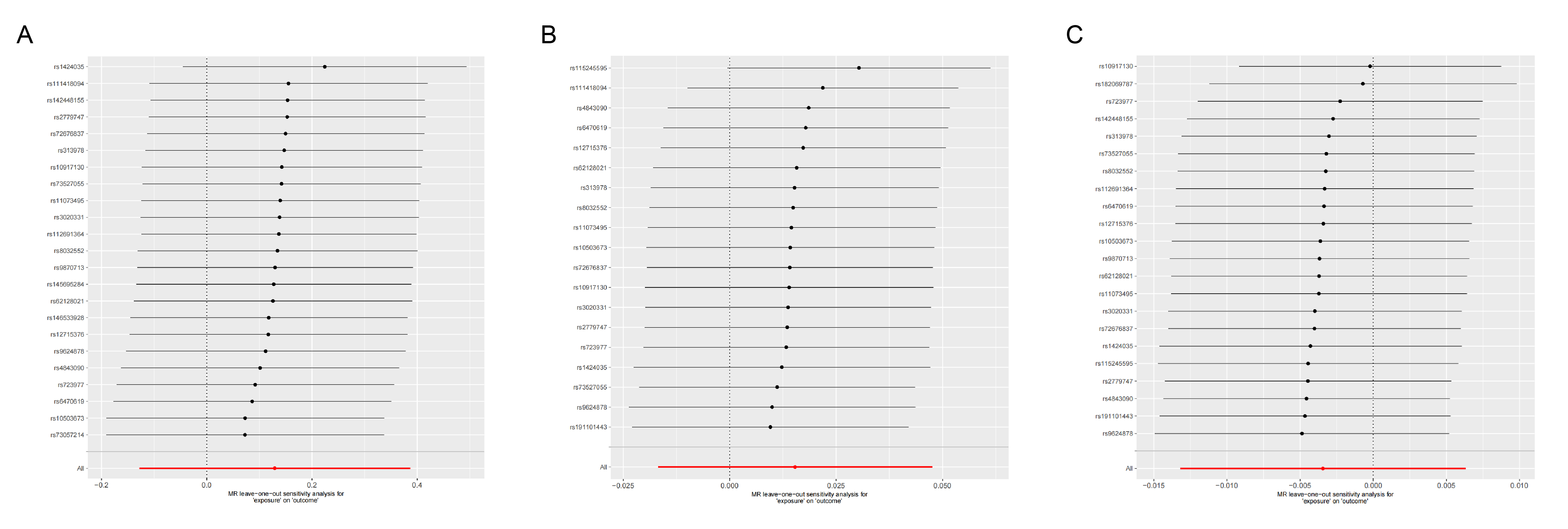

Supplement: Supplementary Figure 8 — The results of ‘leave-one-out’ of mendelian randomization when exposure is female infertility and outcome are mental disorders. (A)-(C) refers the scatter plot of each mental disorders including anxiety disorder, MDD (ieu-b-102), and insomnia. MDD, major depressive disorder; IEU, Integrative Epidemiology Unit. [file Image8.png]
