# Supplementary material for: Common mental disorders and risk of female infertility: a two-sample Mendelian randomization study
Source: Front Endocrinol (Lausanne). 2024 Sep 30;15:1433624. doi: 10.3389/fendo.2024.1433624 (PMC11471633; doi:10.3389/fendo.2024.1433624)
Supplement: Supplementary file 9 [file DataSheet1.docx]

**Table S1. MR analysis results of five mental disorders and female infertility.**

| **Exposures** | **Methods** | **nSNPs** | **beta** | **se** | ***P*** | **OR (95% CI)** |
| --- | --- | --- | --- | --- | --- | --- |
| Anxiety disorder | MR Egger | 11 | -0.0084 | 0.0508 | 0.8720 | 0.992(0.898, 1.095) |
|  | Weighted median |  | -0.0018 | 0.0246 | 0.9402 | 0.998(0.951, 1.047) |
|  | Inverse variance weighted |  | 0.0163 | 0.0183 | 0.3724 | 1.016(0.981, 1.054) |
|  | Simple mode |  | -0.0152 | 0.0345 | 0.6680 | 0.985(0.921, 1.054) |
|  | Weighted mode |  | -0.0080 | 0.0325 | 0.8099 | 0.992(0.931, 1.057) |
| Broad depression | MR Egger | 15 | -0.9365 | 1.8541 | 0.6219 | 0.392(0.010, 14.842) |
|  | Weighted median |  | 0.3707 | 0.7073 | 0.6002 | 1.449(0.362, 5.795) |
|  | Inverse variance weighted |  | 0.6081 | 0.5335 | 0.2543 | 1.837(0.646, 5.227) |
|  | Simple mode |  | 0.4130 | 1.1785 | 0.7312 | 1.511(0.150, 15.224) |
|  | Weighted mode |  | 0.6275 | 1.1288 | 0.5871 | 1.873(0.205, 17.117) |
| MDD (PGC) | MR Egger | 33 | 0.1207 | 0.5063 | 0.8132 | 1.128(0.418, 3.043) |
|  | Weighted median |  | 0.2697 | 0.1201 | 0.0248 | 1.310(1.035, 1.657) |
|  | Inverse variance weighted |  | 0.3337 | 0.0877 | **0.0001** | 1.396(1.176, 1.658) |
|  | Simple mode |  | 0.1657 | 0.2731 | 0.5483 | 1.180(0.691, 2.016) |
|  | Weighted mode |  | 0.1873 | 0.2448 | 0.4498 | 1.206(0.746, 1.949) |
| MDD (ieu-b-102) | MR Egger | 24 | -0.3628 | 0.8403 | 0.6702 | 0.696(0.134 - 3.612) |
|  | Weighted median |  | 0.3641 | 0.1521 | 0.0167 | 1.439(1.068 - 1.939) |
|  | Inverse variance weighted |  | 0.3450 | 0.1112 | **0.0019** | 1.412(1.136 - 1.756) |
|  | Simple mode |  | 0.3088 | 0.3445 | 0.3793 | 1.362(0.693 - 2.675) |
|  | Weighted mode |  | 0.0805 | 0.3288 | 0.8087 | 1.084(0.569 - 2.065) |
| Bipolar disorder | MR Egger | 6 | -0.6760 | 0.4759 | 0.2285 | 0.509(0.200, 1.293) |
|  | Weighted median |  | -0.0457 | 0.1003 | 0.6487 | 0.955(0.785, 1.163) |
|  | Inverse variance weighted |  | -0.1103 | 0.0814 | 0.1758 | 0.896(0.763, 1.051) |
|  | Simple mode |  | 0.0084 | 0.1541 | 0.9589 | 1.008(0.745, 1.364) |
|  | Weighted mode |  | -0.0009 | 0.1481 | 0.9954 | 0.999(0.747, 1.336) |
| Insomnia | MR Egger | 30 | -0.3071 | 0.6102 | 0.6187 | 0.736(0.222, 2.432) |
|  | Weighted median |  | -0.2019 | 0.3429 | 0.5560 | 0.817(0.417, 1.600) |
|  | Inverse variance weighted |  | 0.1081 | 0.2226 | 0.6273 | 1.114(0.720, 1.724) |
|  | Simple mode |  | -0.2561 | 0.5919 | 0.6684 | 0.774(0.243, 2.469) |
|  | Weighted mode |  | -0.2054 | 0.4784 | 0.6708 | 0.814(0.319, 2.080) |

MDD, major depressive disorder; PGC, Psychiatric Genomics Consortium, IEU, Integrative Epidemiology Unit.

The bold *P* - values were ≤0.01.

**Table S2. The heterogeneity test of five mental disorders genetic variants in female infertility Genome-wide summary association study (GWAS) datasets.**

| **Exposures** | **Methods** | **Q** | **Q_df** | **Q_pval** |
| --- | --- | --- | --- | --- |
| Anxiety disorder | MR Egger | 7.814313 | 9 | 0.552962 |
|  | Inverse variance weighted | 8.086953 | 10 | 0.620344 |
| Broad depression | MR Egger | 5.415842 | 13 | 0.964863 |
|  | Inverse variance weighted | 6.17259 | 14 | 0.961952 |
| MDD (PGC) | MR Egger | 36.5781 | 31 | 0.225576 |
|  | Inverse variance weighted | 36.79369 | 32 | 0.256515 |
| MDD (ieu-b-102) | MR Egger | 20.99037 | 17 | 0.226721 |
|  | Inverse variance weighted | 21.2961 | 18 | 0.264739 |
| Bipolar disorder | MR Egger | 3.982955 | 4 | 0.408318 |
|  | Inverse variance weighted | 5.435212 | 5 | 0.365102 |
| Insomnia | MR Egger | 25.85875 | 28 | 0.580809 |
|  | Inverse variance weighted | 26.39271 | 29 | 0.604434 |

MDD, major depressive disorder; PGC, Psychiatric Genomics Consortium, IEU, Integrative Epidemiology Unit.

**Table S9. MR analysis results of female infertility and five mental disorders.**

| **Outcomes** | **Methods** | **nSNPs** | **beta** | **se** | ***P*** | **OR (95% CI)** |
| --- | --- | --- | --- | --- | --- | --- |
| Anxiety disorder | MR Egger | 23 | -0.305 | 0.2888 | 0.303 | 0.737(0.418 - 1.298) |
|  | Weighted median |  | -0.0396 | 0.1868 | 0.8323 | 0.961(0.666 - 1.386) |
|  | Inverse variance weighted |  | 0.1291 | 0.1312 | 0.3252 | 1.138(0.880 - 1.472) |
|  | Simple mode |  | -0.0845 | 0.391 | 0.831 | 0.919(0.427 - 1.978) |
|  | Weighted mode |  | -0.121 | 0.3853 | 0.7565 | 0.886(0.416 - 1.886) |
| Broad depression | Wald ratio | 1 | 0.0223 | 0.0181 | 0.2192 | 1.022(0.987 - 1.060) |
| MDD (PGC) | Inverse variance weighted | 2 | 0.0335 | 0.0474 | 0.4798 | 1.034(0.942 - 1.135) |
| MDD (ieu-b-102) | MR Egger | 19 | 0.0016 | 0.0323 | 0.9604 | 1.002(0.940 - 1.067) |
|  | Weighted median |  | 0.0394 | 0.0217 | 0.0692 | 1.040(0.997 - 1.085) |
|  | Inverse variance weighted |  | 0.0154 | 0.0164 | 0.3489 | 1.016(0.983 - 1.049) |
|  | Simple mode |  | 0.0446 | 0.0371 | 0.2451 | 1.046(0.972 - 1.125) |
|  | Weighted mode |  | 0.0439 | 0.0336 | 0.2073 | 1.045(0.978 - 1.116) |
| Bipolar disorder | Wald ratio | 1 | -0.0608 | 0.2114 | 0.7735 | 0.941(0.622 - 1.424) |
| Insomnia | MR Egger | 22 | -0.0022 | 0.0079 | 0.7802 | 0.998(0.983 - 1.013) |
|  | Weighted median |  | 0.0011 | 0.0065 | 0.8684 | 1.001(0.988 - 1.014) |
|  | Inverse variance weighted |  | -0.0034 | 0.005 | 0.4876 | 0.997(0.987 - 1.006) |
|  | Simple mode |  | 0.0003 | 0.0112 | 0.9756 | 1.000(0.979 - 1.023) |
|  | Weighted mode |  | 0.0037 | 0.0112 | 0.745 | 1.004(0.982 - 1.026) |

MDD, major depressive disorder; PGC, Psychiatric Genomics Consortium, IEU, Integrative Epidemiology Unit.

The bold *P* - values were ≤0.01.

**Table S10. The heterogeneity test of female infertility genetic variants in five mental disorders Genome-wide summary association study (GWAS) datasets.**

| **Outcomes** | **Methods** | **Q** | **Q_df** | **Q_pval** |
| --- | --- | --- | --- | --- |
| Anxiety disorder | MR Egger | 18.4974 | 21 | 0.617347 |
|  | Inverse variance weighted | 21.34402 | 22 | 0.499573 |
| MDD (PGC) | Inverse variance weighted | 0.028359 | 1 | 0.866268 |
| MDD (ieu-b-102) | MR Egger | 20.99037 | 17 | 0.226721 |
|  | Inverse variance weighted | 21.2961 | 18 | 0.264739 |
| Insomnia | MR Egger | 25.8293 | 20 | 0.171524 |
|  | Inverse variance weighted | 25.88207 | 21 | 0.210982 |

MDD, major depressive disorder; PGC, Psychiatric Genomics Consortium, IEU, Integrative Epidemiology Unit.
